# Deletion of a gene cluster encoding pectin degrading enzymes in *Caldicellulosiruptor bescii* reveals an important role for pectin in plant biomass recalcitrance

**DOI:** 10.1186/s13068-014-0147-1

**Published:** 2014-10-10

**Authors:** Daehwan Chung, Sivakumar Pattathil, Ajaya K Biswal, Michael G Hahn, Debra Mohnen, Janet Westpheling

**Affiliations:** Department of Genetics, University of Georgia, Athens, GA 30602 USA; Department of Biochemistry and Molecular Biology, Complex Carbohydrate Research Center, University of Georgia, Athens, GA 30602 USA; The BioEnergy Science Center, Oak Ridge National Laboratory, Oak Ridge, TN 37831 USA; Department of Plant Biology, University of Georgia, Athens, GA 30602 USA

**Keywords:** Bioenergy, Biomass deconstruction, Pectin, Thermophile

## Abstract

**Background:**

A major obstacle, and perhaps the most important economic barrier to the effective use of plant biomass for the production of fuels, chemicals, and bioproducts, is our current lack of knowledge of how to efficiently and effectively deconstruct wall polymers for their subsequent use as feedstocks. Plants represent the most desired source of renewable energy and hydrocarbons because they fix CO_2_, making their use carbon neutral. Their biomass structure, however, is a barrier to deconstruction, and this is often referred to as recalcitrance. Members of the bacterial genus *Caldicellulosiruptor* have the ability to grow on unpretreated plant biomass and thus provide an assay for plant deconstruction and biomass recalcitrance.

**Results:**

Using recently developed genetic tools for manipulation of these bacteria, a deletion of a gene cluster encoding enzymes for pectin degradation was constructed, and the resulting mutant was reduced in its ability to grow on both dicot and grass biomass, but not on soluble sugars. The plant biomass from three phylogenetically diverse plants, Arabidopsis (a herbaceous dicot), switchgrass (a monocot grass), and poplar (a woody dicot), was used in these analyses. These biomass types have cell walls that are significantly different from each other in both structure and composition. While pectin is a relatively minor component of the grass and woody dicot substrates, the reduced growth of the mutant on all three biomass types provides direct evidence that pectin plays an important role in biomass recalcitrance. Glycome profiling of the plant material remaining after growth of the mutant on Arabidopsis biomass compared to the wild-type revealed differences in the rhamnogalacturonan I, homogalacturonan, arabinogalactan, and xylan profiles. In contrast, only minor differences were observed in the glycome profiles of the switchgrass and poplar biomass.

**Conclusions:**

The combination of microbial digestion and plant biomass analysis provides a new and important platform to identify plant wall structures whose presence reduces the ability of microbes to deconstruct plant walls and to identify enzymes that specifically deconstruct those structures.

**Electronic supplementary material:**

The online version of this article (doi:10.1186/s13068-014-0147-1) contains supplementary material, which is available to authorized users.

## Background

A fundamental understanding of the physical and chemical structures of plant cell walls is essential to guide the development of processes for biomass deconstruction and to use plant biomass as a feedstock for the production of fuels, chemicals, and bioproducts [1]. Most efforts targeted at improving the deconstruction of plant biomass for biofuel production have centered on crystalline cellulose, lignin, and xylan (the major hemicellulose in grass (for example, switchgrass) walls and in dicot (for example, poplar wood) secondary walls) [2]. Indeed, most models of biomass used in the biofuels field do not list pectin because of its low abundance in grass walls and in dicot secondary walls [3]. Recent work, however, has shown that pectin is synthesized in secondary walls [4], that some pectin biosynthetic enzymes are amplified in grasses [5], and that saccharification of plant biomass can be improved by modifying the structure of pectin [6]. Two recent discoveries provide possible explanations for these observations. Although pectin is present at low levels in secondary walls, which represent the bulk of plant material, the overexpression of a pectin-degrading enzyme during the onset of secondary wall formation in transgenic aspen resulted in increased solubility of pectins and hemicelluloses and resulted in higher yields of pentoses and hexoses, suggesting that, albeit a minor structural component, pectins are important for secondary cell wall architecture [7]. Furthermore, the recent identification of a novel pectin-containing proteoglycan structure [8] in plant walls suggests that current models of the plant cell wall are incomplete, and that the conceptual framework that drives current strategies for overcoming the recalcitrance of biomass for deconstruction and bioproduct formation may need revision. These recent discoveries suggest that pectin-containing structures exist in all plant biomass and that modification of these structures, or at least the pectin domains therein, can be used to decrease biomass recalcitrance.

**Figure 1:**
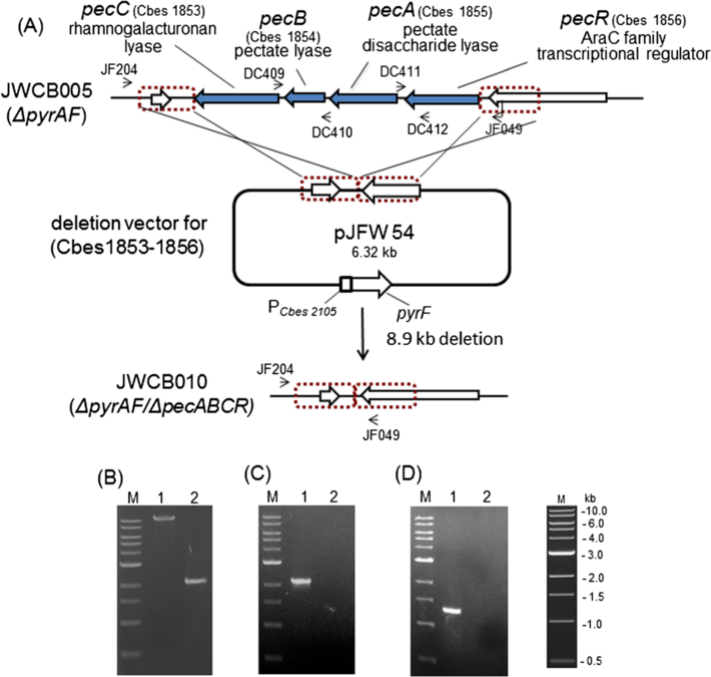
**Strategy for obtaining a deletion of the pectinase gene cluster (Cbes1853-1856) and PCR analysis of the deletion in strain JWCB010. (A)** The genome region containing the cluster with the deletion vector (pJFW54) containing about 2 kb flanking regions from up- and downstream of the cluster and the *pyrF* cassette [18]. Homologous recombination may occur at either the upstream or downstream flanking region, shown within the dotted-line box, integrating the plasmid into the genome and generating a strain that is a uracil prototroph. Counter-selection on 5-FOA selects for loss of the integrated plasmid and possible deletion of the pectinase gene cluster. Black arrows depict primers used for verification of the deletion. **(B)** Gel depicting PCR products of the pectinase gene cluster region in JWCB010 (2.1 kb), the deletion strain (lane 2), compared to its parent, JWCB005 (11 kb) (lane 1), using primers JF049 and JF204. JF204 anneals to a site outside of the homologous regions in the plasmid. **(C)** Gel depicting the 2.18 kb PCR product amplified using primer set (DC409/DC410) from the genome region that includes Cbes1854, *pecB,* JWCB005 (lane 1) or the deletion mutant (lane 2). **(D)** Gel depicting the 1.31 kb PCR product amplified using primer set (DC411/DC412) from the genome region that includes Cbes1855, *pecA* and Cbes1856, *pecR* from the JWCB005 (lane 1) or the deletion mutant (lane 2). M: 1 kb DNA ladder (NEB).

Members of the genus *Caldicellulosiruptor* have the ability to grow on unpretreated biomass, and different species differ in this ability. *C. bescii* is the most thermophilic cellulolytic bacteria (T_opt_ = about 80°C) so far described, and is able to utilize a wide range of substrates such as cellulose, hemicellulose, and diverse types of unpretreated plant biomass [9,10]. The *C. bescii* genome contains a total of five genes predicted to be involved in pectin deconstruction/utilization [11]. Three exist in a single cluster/operon with a predicted transcriptional regulator (Figure 1A), and expression of this cluster is significantly up-regulated in cells growing on biomass [12,13]. The three genes in this cluster encode members of different families of polysaccharide lyases (PLs). *pecA* (Cbes1855) is a PL9, *pecB* (Cbes1854) is a PL3, and *pecC* (Cbes1853) is a PL11. These three multidomain pectinases also contain two kinds of carbohydrate-binding modules (CBMs), CBM3 (*pecC*) and CMB_4_9 (*pecA* and *pecB*), presumably to facilitate binding and degradation of pectin or other compounds in plant biomass (Table 1). Cbes1853 was previously annotated as a cellobiohydrolase in GenBank (http://www.ncbi.nlm.nih.gov/genbank/). Our own bioinformatic analysis of the predicted protein sequence using BLASTn [14] and BLASTx [15] suggests that it is more likely a rhamnogalacturonan lyase [16]. Only *C. bescii* and *C. kristjansonii*, of the eight *Caldicellulosiruptor* species sequenced, contain all three genes in the cluster [9,11]. The two other genes predicted to be involved in pectin degradation are Cbes2380, annotated as a glycoside hydrolase family 28, galacturan 1,4-alpha-galacturonidase [11], and Cbes2353, annotated as a hypothetical protein with sequence homology to rhamnogalacturonan lyase of the plysaccharide lyase family 11 [12]. While there is no direct biochemical evidence for the enzymatic activity of most of the proteins encoded by these genes, Cbes1854, *pecB*, was cloned and expressed in *E. coli* and was shown to have pectate lyase activity [17].

**Table 1:** **Genes in the predicted pectinase gene cluster in** ***Caldicellulosiruptor bescii***

**Gene**	**Predicted gene product**	**CAZy module architecture**	**Gene location/size**
*pecA* (Cbes1855)	Pectate disaccharide lyase (EC:4.2.2.9)	SP-CBM_4_9-PL9	1933527-1935488/1.962 kb
*pecB* (Cbes1854)	Pectate lyase (EC:4.2.2.2)	SP-CBM_4_9-PL3	1932088-1933470/1.383 kb
*pecC* (Cbes1853)	Rhamnogalacturonan lyase	SP-PL11-CBM3	1929397-1931898/2.502 kb
*pecR* (Cbes1856)	AraC family transcriptional regulator		1935834-1938215/2.382 kb

Cbes1853, 1854, and 1855 are predicted to encode extracellular CAZy proteins [19] which contain signal peptides at the 5’- end and a CBM. The abbreviations are: CAZy, carbohydrate active enzyme; EC, enzyme commission number; SP, signal peptide; PL3, PL9, and PL11, polysaccharide lyase family of 3, 9, and 11; CBM, carbohydrate-binding module; CBM3, carbohydrate-binding module of family 3; CBM_4_9, putative pectin-binding module [12,13].

Using recently developed tools for genetic manipulation of *C. bescii* [20,21], a deletion of the pectinase gene cluster was constructed in the *C. bescii* genome, resulting in a mutant that is reduced in its ability to grow on both dicot and grass biomass. The phenotype of the *C. bescii* pectinase mutant provides direct genetic evidence that pectin is a significant barrier to deconstruction of unpretreated plant biomass by *C. bescii* and that removal of pectin is essential for maximum deconstruction of plant biomass by *C. bescii.* Glycome profiling of the plant cell walls before and after growth of *C. bescii* and the pectinase mutant, using cell wall glycan-directed monoclonal antibodies (mAbs), revealed structural changes, thereby providing some insight into the function of the pectinase gene cluster in plant biomass deconstruction. Glycome profiling analyses revealed differential utilization of Arabidopsis, switchgrass, and poplar biomass by these microbes, likely due to the variations in cell wall structure and composition among them.

## Results and discussion

### Deletion of a pectinase gene cluster in the *C. bescii* chromosome

To investigate the role of the pectinase gene cluster in the deconstruction of unpretreated plant biomass by *C. bescii*, we constructed a deletion of this cluster in the *C. bescii* chromosome by marker replacement (Figure 1A). The cluster includes three pectate lyases, *pecA, pecB, pecC* (Cbes1853-1855), and a putative AraC family transcriptional regulator, designated *pecR* (Cbes1856). The deletion was constructed on a non-replicating plasmid, pJFW54, by fusing the 5’ and 3’ flanking regions of the gene cluster (Figure 1A). The plasmid also contained a wild-type copy of the *pyrF* gene under the transcriptional control of the ribosomal protein S30EA (Cbes2105) promoter (Additional file 1: Figure S1) [18] for selection of transformants. pJFW54, isolated from *E. coli,* was methylated in vitro with M.CbeI methyltransferase [20] and electrotransformed into strain JWCB005 (Table 2), which contained a deletion of the *pyrFA* locus. Transformants were selected for uracil prototrophy. Uracil prototrophy resulted from a single crossover event integrating the plasmid at the flanking region of the targeted pectinase cluster locus. Integration at the targeted region was confirmed by PCR amplification of the region and sequencing of the PCR products. Cells were then plated on a minimal medium containing 5-fluoroorotic acid (5-FOA) and uracil to counter-select the wild-type *pyrF* allele, requiring a second crossover event to eliminate the plasmid from the chromosome restoring uracil auxotrophy. Integration of the plasmid at the pectinase gene cluster locus and excision of the plasmid resulting in deletion of the cluster was confirmed by PCR amplification analysis of the region (Figure 1B,C, and D) and DNA sequencing of the PCR products.

**Table 2:** **Strains and plasmids used in this study**

**Strain/plasmid**	**Strain and genotype/phenotype**	**Source**
DSM6725	*C. bescii* wild-type (ura^+^/5-FOsA^S^)	DSMZ^1^
JWCB005	*C. bescii ΔpyrFA* (ura^−^/5-FOA^R^)	[18]
JWCB010	JWCB005 *ΔpyrFA ΔpecABCR* (ura^−^/5-FOA^R^)	This study
pDCW88	*cbe1* deletion vector (Apramycin^R^)	[21]
pJFW54	*pecABCR* deletion vector (Apramycin^R^)	This study

^*1*^ *German Collection of Microorganisms and Cell Culture.*

Isolates containing clean deletions of the cluster were further purified on solid medium without 5-FOA, and the deletion was confirmed by PCR analysis using primers located outside the homologous regions used to generate the deletions (Figure 1B). PCR amplification of the pectinase gene cluster genome region in JWCB005 produced an approximately 11-kb band, while a band of about 2.1 kb was amplified from JWCB010 (*ΔpyrFA, ΔpecABCR)*, as predicted (Table 1). The pectinase gene cluster is 8.9 kb (Figure 1A). The absence of pectinase genes in the cluster was further confirmed by PCR with primer pairs targeting *pecA, pecB,* and *pecR* within the cluster (Figure 1C and D) to eliminate the possibility of contamination by merodiploids resulting from incomplete genome segregation. The approximately 2.2- and 1.3-kb products were amplified from the parental strain (JWCB005), but not from the deletion strain (JWCB010). A real-time PCR analysis was performed to test whether the deletion affected the expression of the surrounding genes (Cbes1852 and Cbes1857), and there was no effect on their expression by the deletion (data not shown).

### Deletion of the pectinase gene cluster does not affect growth on the soluble substrates maltose and cellobiose

To examine the effect of the *pecABCR* deletion on the growth of *C. bescii* on diverse substrates, we first tested its ability to grow on maltose and cellobiose, simple soluble substrates that do not induce increased expression of the gene cluster [12,13]. While the growth properties of the different *Caldicellulosiruptor* strains on various sugar substrates have been reported in several studies [10–12,22], these analyses were performed in modified DSMZ medium 640 which includes 0.05% (w/v) yeast extract (YE). This amount of YE supports growth of *C. bescii* in the absence of an additional carbon source [23], and may well stimulate growth on poor carbon sources such as complex plant biomass, thereby complicating the analyses and their interpretation. We recently reported the formulation of a defined low osmolarity minimal medium, LOD [23], and used this medium exclusively in the analyses of growth on various carbon sources reported in this work.

**Figure 2:**
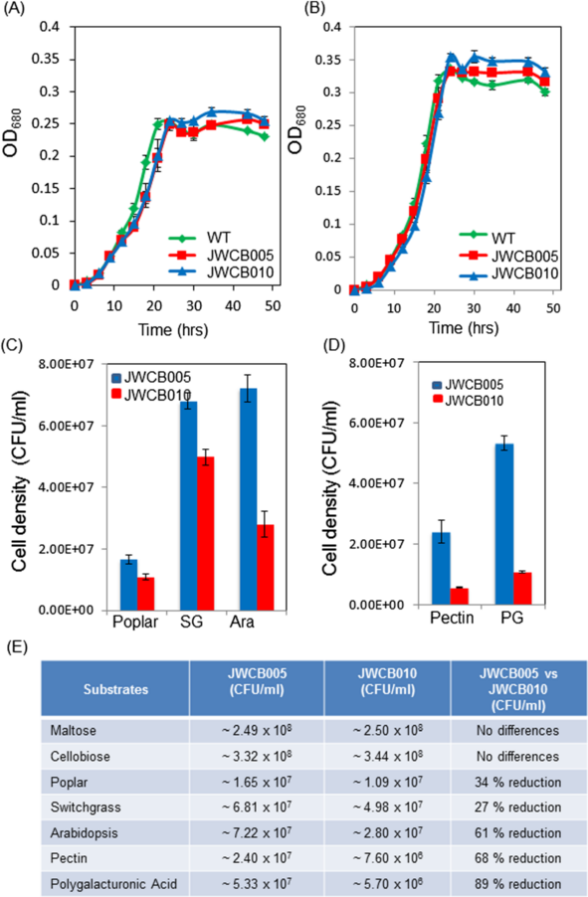
**Analysis of growth of the pectinase gene cluster deletion mutant (JWCB010) on selected substrates.** All substrates were provided at 0.5 g/L in LOD medium [23]. Growth curves and histograms represent the average ± SD of at least three biological replicates. Substrate abbreviations: SG, switchgrass; Ara, Arabidopsis; PG, polygalacturonic acid. CFU stands for colony-forming unit. Comparison of growth of JWCB005 (*ΔpyrFA*) and JWCB010 (*ΔpyrFA ΔpecABCR*) on **(A)** maltose, **(B)** cellobiose, **(C)** plant biomass, and **(D)** pectin compounds. **(E)** Differences in growth on various substrates*.* Strains were grown on the designated carbon source for 24 h at 75°C. For each comparison *P* < 0.012.

We performed a direct comparison of the growth properties of wild-type *C. bescii*, JWCB005 (*C. bescii ΔpyrFA)* and JWCB010 (*C. bescii ΔpyrFA ΔpecABCR)* on different substrates to investigate the effect of the absence of this pectinase gene cluster on carbon utilization by *C. bescii*. Strains were grown in triplicate on all substrates tested. For the soluble substrates maltose and cellobiose, growth was measured spectrophotometrically at OD_680_ (Figure 2A,B). Cells grew abundantly on both maltose (Figure 2A, Additional file 1: Figure S2A) and cellobiose (Figure 2B) and there was virtually no difference in growth between the wild-type, the parent strain, and the pectinase cluster deletion mutant. This was expected since maltose and cellobiose are simple, soluble carbohydrates that do not induce expression of the gene cluster. These data suggest that there is no general defect in the growth of either the parent strain (*ΔpyrFA)* or the pectinase deletion mutant (*ΔpyrFA ΔpecABCR)* compared to the wild-type. They also suggest that the pectinase gene cluster is not required to fully utilize these substrates.

### Deletion of the pectinase gene cluster results in a defect in growth of *C. bescii* on complex carbohydrates and plant biomass

To compare growth of the *C. bescii* parent strain JWCB005 (*ΔpyrFA*) and the pectinase cluster deletion mutant JWCB010 (*ΔpyrFA ΔpecABCR*) on complex substrates, the two strains were grown on five different complex substrates: unpretreated aerial switchgrass (SG), seven-week-old aerial Arabidopsis (Ara) tissue, and unpretreated poplar wood, pectin, and polygalacturonic acid (PG). We emphasize that the biomass substrates were not autoclaved prior to their use as growth substrates. The biomass was ground, added to a minimal salts medium, LOD [23], inoculated with cells, and heated to 75°C, the optimal growth temperature of *C. bescii*. The cell densities were measured after 24 h at 75°C (Figure 2C,D). Growth for 24 h corresponds to the mid-log phase. Growth on insoluble substrates was measured by direct cell counts. Optical density is accurate for assessing growth on soluble substrates, but not for assessing growth on complex insoluble substrates such as biomass, since cells adhere loosely to the particulate biomass matrix and cannot be distinguished from cell debris under a light microscope. To obtain accurate cell numbers during growth on biomass, cells were stained with 0.1% (w/v) acridine orange and visualized with an Olympus BX40 phase contrast microscope with an oil-immersion objective lens containing a cell counting objective (Uplan F1 100X/1.3) [9]. As shown in Figures 2D and E, growth of the pectinase deletion mutant on pectin and polygalacturonic acid was reduced 68% and 89%, respectively, compared to growth of the parent strain on the same substrates. The reduced growth of the deletion mutant on the pectins supports the predicted activity of the enzymes in the gene cluster in pectin degradation.

These results indicate that the predicted pectate lyases and rhamnogalacturonan lyases encoded by the gene cluster were required to obtain optimal growth on the pectic substrates. Perhaps more interestingly, however, the growth of the mutant *C. bescii* was reduced 61% on Arabidopsis, 27% on switchgrass, and 34% on poplar (Figure 2C,E and Additional file 1: Figure S2). The greater reduction of growth of the *C. bescii* pectinase gene cluster mutant on Arabidopsis compared to switchgrass and poplar biomass is likely because, as an herbaceous dicot, Arabidopsis vegetative biomass contains largely primary cell walls with 30 to 35% pectin compared to the 2 to 10% pectin in switchgrass and poplar biomass walls [3]. However, the amount of reduced growth on all three biomass sources is significantly greater than that expected from only deconstruction and metabolism of the pectin present on a percent mass level in the different biomass types. Rather, the results suggest that deconstruction of pectin moieties in the plant biomass may have enabled *C. bescii* to gain access to, and metabolize, the other polymers in the wall. To test this hypothesis, the biomass remaining after growth of *C. bescii* was analyzed by an immunological technique known as glycome profiling [24,25].

### Glycome profiling of biomass remaining after growth of the *C. bescii* wild-type, JWCB005 strain, and pectinase deletion mutant JWCB010

The effect of the deletion of the pectinase gene cluster on deconstruction and metabolism of the different types of biomass was examined by fingerprinting cell wall preparations from the phylogenetically diverse Arabidopsis (a herbaceous dicot), switchgrass (a monocot grass), and poplar (a woody dicot) biomass using glycome profiling to detect the presence of diverse immunologically reactive glycan epitopes. These three biomass types were incubated with the wild-type strain, the parent strain JWCB005 (*ΔpyrFA*), and the mutant strain JWCB010 (*ΔpyrFA ΔpecABCR)*, and the solid residues remaining after microbial incubation were used for glycome profiling.

**Figure 3:**
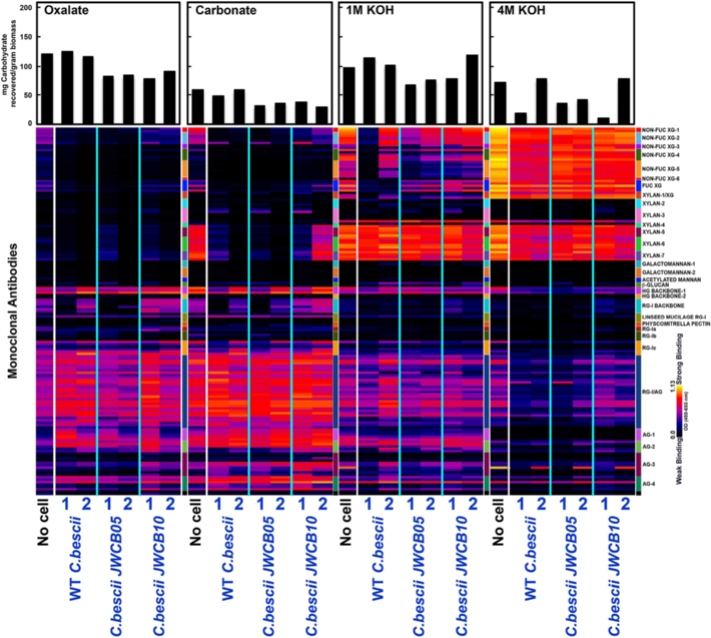
**Glycome profiling of Arabidopsis biomass samples before and after incubation with wild-type** ***C.bescii*** **, JWCB005 (** ***ΔpyrFA*** **), and JWCB010 (** ***ΔpyrFA ΔpecABCR*** **).** Sequential cell wall extracts were prepared from biomass recovered after 24 hr of growth at 75°C of the designated *C. bescii* strains. The cell walls were isolated as alcohol insoluble residues (AIRs) from the designated biomass, and the AIR was extracted sequentially using ammonium oxalate (oxalate), sodium carbonate and potassium hydroxide (1 M KOH and 4 M KOH) as described in the Methods section. The extracts were screened by ELISA using 155 mAbs directed against diverse epitopes present on most plant cell wall glycans (Additional file 1: Table S1). The resulting binding response data are represented as heatmaps with white-red-dark blue scale indicating the strength of the ELISA signal (white, red, and dark-blue colors depict strong, medium, and no binding, respectively). The mAbs are grouped based on the cell wall glycans they recognize as depicted in the panel at the right-hand side of the figure. The actual amounts of materials extracted in each extraction step are depicted as bar graphs at the top of the heatmaps. The data represent the average of two biological replicates. The soluble material in the original growth media was also analyzed (Additional file 1: Figure S3).

Glycome profiles of cell walls from Arabidopsis biomass incubated without bacterial cells differed significantly from the biomass remaining after incubation with *C. bescii* cells (Figure 3). Specifically, a significant difference was noted in the carbonate extracts. Xylan epitopes were abundant in the carbonate extract from walls incubated without bacteria, while these epitopes were almost totally absent in the samples incubated with wild-type *C. bescii* or with the parent strain. However, when considering the averaged trend, incubation of the biomass with the *C. bescii* pectinase gene cluster mutant resulted in retention of some xylan epitopes in the carbonate extracts, unlike the results with the wild-type or parent strain. Another significant difference was noted in oxalate and carbonate extracts of biomass, where extracts of walls incubated with wild-type *C. bescii* and the parent strain showed an almost total absence of rhamnogalacturonan I (RG-I) backbone epitopes. In contrast, these epitopes were clearly present in the corresponding oxalate and carbonate extracts from biomass to which no bacteria had been added and in biomass incubated with the pectinase deletion mutant. These results suggest a possible function for the pectinase gene cluster in the metabolism of the RG-I backbone. A similar observation was made in the case of homogalacturonan backbone-1, RG-I backbone, and pectic-arabinogalactan epitopes in the 4 M KOH extracts, where a significant reduction in the abundance of these epitopes was seen only in the samples incubated with wild-type *C. bescii* or the parent strain. These data suggest that wild-type *C. bescii* and the parent strain, both containing an intact copy of the pectinase gene cluster, utilize xylan and pectin more efficiently when growing on Arabidopsis biomass than the pectinase deletion strain. Another effect of the growth of all three strains on the Arabidopsis biomass was a significantly reduced abundance of some xyloglucan epitopes (recognized by the Non-Fuc XG-5 set of antibodies) in the 1 M KOH extracts, suggesting that these strains may potentially utilize a subpopulation of hemicellulosic xyloglucans in Arabidopsis biomass. Furthermore, the deletion mutant less effectively utilized another subpopulation of xyloglucan (recognized by the Non-Fuc XG-4 and Fuc-XG groups of antibodies) compared with the WT and parent strains. Finally, there are also differences in the pectic arabinogalactan and arabinogalactan epitopes released into the medium by the three microbes. Specifically, the epitopes recognized by the RG-I/AG antibodies JIM132, JIM1, CCRC-M15, and CCRC-M8 and the AG-3 antibodies JIM15 and JIM8 were absent in the media supernatant from the mutant (Additional file 1: Figure S3).

**Figure 4:**
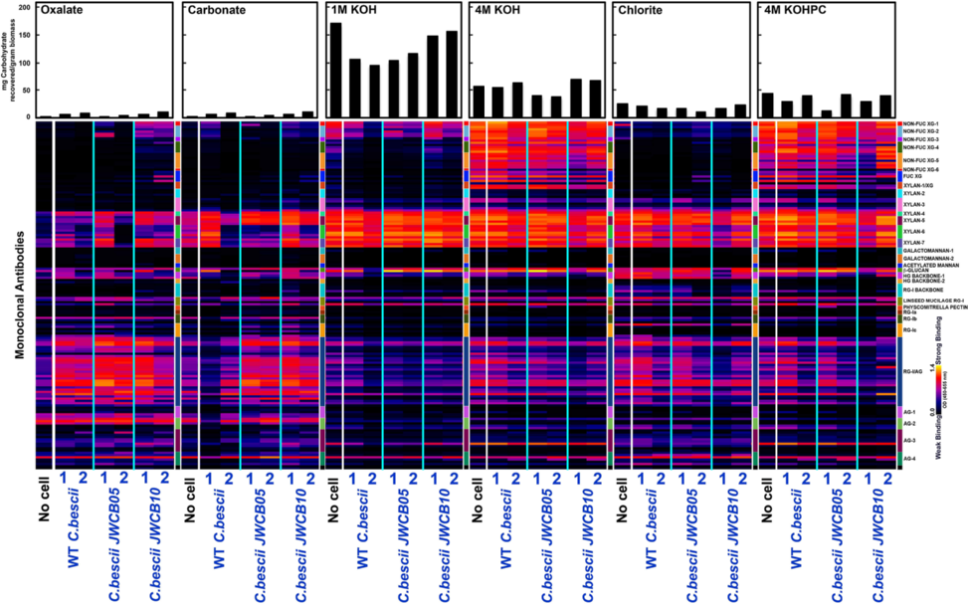
**Glycome profiling of switchgrass biomass samples before and after incubation with wild-type** ***C.bescii*** **, JWCB005 (** ***ΔpyrFA*** **), and JWCB010 (** ***ΔpyrFA ΔpecABCR*** **).** The cell walls were isolated as alcohol insoluble residues (AIRs), and the the AIR was extracted sequentially using ammonium oxalate (oxalate), sodium carbonate, and potassium hydroxide (1 M KOH, 4 M KOH, and 4 M KOHPC) and sodium chlorite (NaClO_4_) as described in the Methods section. The extracts were screened by ELISA using 155 mAbs directed against diverse epitopes present on most plant cell wall glycans (Additional file 1: Table S1). The resulting binding response data are represented as heatmaps with white-red-dark blue scale indicating the strength of the ELISA signal (white, red, and dark-blue colors depict strong, medium, and no binding, respectively). The mAbs are grouped based on the cell wall glycans they recognize as depicted in the panel at the right-hand side of the figure. The data represent the average of two biological replicates. The actual amounts of materials extracted in each extraction step are depicted as bar graphs at the top of the heatmaps. The soluble material is the original growth medium was also analyzed (Additional file 1: Figure S4).

Analyses of switchgrass biomass (Figure 4) showed that incubation with all three strains invariably caused changes in the glycan epitopes extractable from the biomass. For example, incubation with all three strains resulted in an increased extractability of pectic arabinogalactan epitopes in the oxalate extracts. In carbonate extracts, an increase in the extractability of pectic arabinogalactan epitopes was evident but more consistently in the biomass incubated with the parent and deletion mutant strains. Again, a general increased abundance of oxalate-extractable xylan epitopes was noted in the microbe-incubated biomass. Switchgrass biomass incubated with the deletion strain released consistently greater amounts of carbohydrate material in the 1 M KOH and 4 M KOH extracts in comparison with those incubated with either the wild-type or parent strains (see the top bar graphs in Figure 4). In addition, analysis of the growth media after removal of the cells using the 155 monoclonal antibodies in the toolkit revealed the presence and/or greater abundance of xylan, homogalacturonan, rhamnogalacturonan, and arabinogalactan epitopes in the medium from the pectinase gene cluster mutant compared to that from the wild-type or the parent strain (Additional file 1: Figure S4), suggesting a reduced ability to break down these polysaccharides by the deletion mutant.

**Figure 5:**
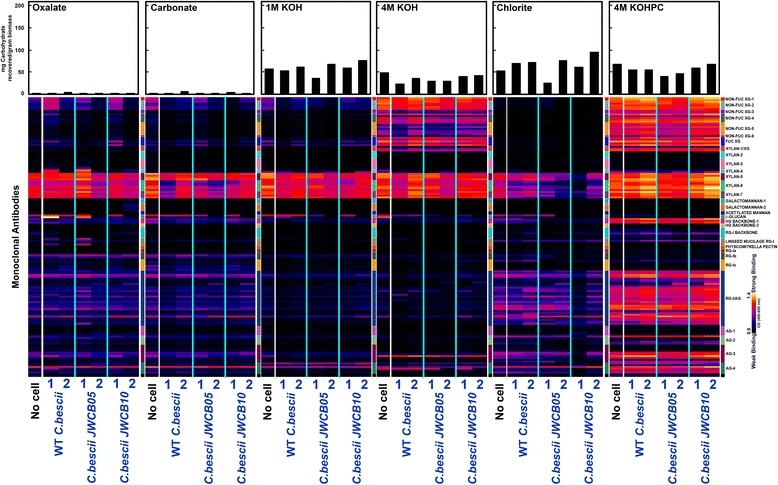
**Glycome profiling of poplar biomass samples before and after incubation with wild-type** ***C.bescii*** **, JWCB005 (** ***ΔpyrFA*** **), and JWCB010 (** ***ΔpyrFA ΔpecABCR*** **).** The cell walls were isolated as alcohol insoluble residues (AIRs), and the AIR was extracted sequentially using ammonium oxalate (oxalate), sodium carbonate, and potassium hydroxide (1 M KOH, 4 M KOH, and 4 M KOHPC) and sodium chlorite (NaClO_4_) as described in the Methods section. The extracts were screened by ELISA using 155 mAbs directed against diverse epitopes present on most plant cell wall glycans (Additional file 1: Table S1). The resulting binding response data are represented as heatmaps with white-red-dark blue scale indicating the strength of the ELISA signal (white, red, and dark-blue colors depict strong, medium, and no binding, respectively). The mAbs are grouped based on the cell wall glycans they recognize as depicted in the panel at the right-hand side of the figure. The data represent the average of two biological replicates. The actual amounts of materials extracted in each extraction step are depicted as bar graphs at the top of the heatmaps.

Analyses of poplar biomass (Figure 5) showed that, during their growth, all three strains induced lesser changes in their cell walls in comparison to Arabidopsis and switchgrass. Consistent with the significant abundance of secondary walls in poplar biomass (and hence a higher abundance of lignin), the chlorite extracts of poplar biomass released significantly greater amounts of cell wall carbohydrate material in comparison with switchgrass. Interestingly, in chlorite extracts, poplar biomass incubated with the deletion mutant showed marginally less pectic-arabinogalactan and xylan epitopes than those incubated with wild-type *C. bescii* or the parent strain. These data suggest that the three strains differentially utilize lignin-associated arabinogalactan-rich glycans from Arabidopsis, switchgrass, and poplar biomass.

Taken together, these results show that a deletion of the pectinase gene cluster resulted in a strain that was less efficient in utilizing pectic components in Arabidopsis, switchgrass, and poplar biomass compared to the wild-type *C.bescii* and the parent strain. The results also show that the pectinase deletion mutant differs in its ability to release some xylan, arabinogalactan, xylogucan, and homogalacturonan glycan epitopes from the biomass.

## Conclusions

Data from both microbial biomass deconstruction experiments and plant cell wall biosynthesis studies demonstrate that one or more pectin structures limit accessibility of microbes and/or microbial enzymes to plant biomass; that is, one or more pectic structures are a recalcitrance barrier to biomass deconstruction. We propose that knowledge of the pectin structures that affect recalcitrance and the means to degrade the structure(s) offer a way to improve biomass deconstruction and to provide technology to increase the cost-effectiveness of using biomass for fuels and biomaterials production. Since grasses and woody species in general contain only a small percentage of their mass as pectin, it was surprising that the deletion of a gene cluster encoding pectin-degrading enzymes had such a dramatic effect on the ability of *C. bescii* to use switchgrass and poplar as a carbon source. These results suggest a more critical role for pectin in biomass recalcitrance than generally appreciated. Furthermore, we suggest that a combined microbe/plant biomass platform to identify plant wall structures whose presence reduces the ability of microbes to deconstruct plant walls will identify enzymes that deconstruct those structures. Indeed, prior work has demonstrated the utility of using plant cell wall degrading enzymes to probe plant cell wall structure and to provide data for constructing cell wall models [26], as well as to characterize wall polymers affected in specific plant cell wall mutants [27]. The approach described in this paper has the potential to combine plant and microbial genetics and biomass chemistry to guide the development of a process to achieve efficient energy harvesting and conversion. Overexpression of pectin utilization genes in microbes may increase their cellulolytic ability and might facilitate conversion by new microbial hosts. A process that takes advantage of the natural components of plants and microbes and does not use chemicals or added enzymes for pretreatment and without generating toxic products would also be environmentally benign.

## Methods

### Strains, media, and growth conditions

The *Caldicellulosiruptor bescii* strains and the plasmid used in this study are listed in Table 2. All *C. bescii* strains were grown anaerobically in liquid or solid medium in LOD medium [23] with maltose at 75°C as previously described for routine growth and transformation experiments [21]. For the growth of uracil auxotrophs JWCB005 (*ΔpyrFA*) and JWCB010 (*ΔpyrFA ΔpecABCR*), the defined medium contained 20 μM uracil. *E. coli* strain DH5α was used for plasmid DNA constructions and preparation. Standard techniques for *E. coli* were performed as described [28]. *E. coli* cells were grown in LB broth supplemented with apramycin (50 μg/mL) and plasmid DNA was isolated using a Qiagen Mini-prep Kit. Genomic DNA from *Caldicellulosiruptor* species was extracted using the Quick-gDNA™ MiniPrep (Zymo, Irvine, CA) according to the manufacturer’s instructions.

### Plant materials and growth conditions

Wild-type *Arabidopsis thaliana* ecotype Columbia (S6000) seeds were sequentially treated with 70% (v/v) EtOH for 1 min and 10% (v/v) bleach (Clorox) for 10 min, and sterilized with ddH_2_O eight to ten times. The sterilized seeds were plated on ½ Murashige and Skoog (MS) media plates (pH 5.7) containing 5.5 g/L plant agar (Research Products International Corp, Mount Prospect, IL) and germinated in a Conviron growth chamber under 14 h light/8 h dark photoperiod at 22°C, 60% relative humidity, and a light intensity of 150 μmol photons m^-2^ s^-1^. Two-week-old seedlings were transferred from plates to premoistened soil in a growth chamber with 50% constant relative humidity and a photoperiod 14/10 light/dark cycle (14 h 19°C and 10 h 15°C), and were fertilized as described [29]. Aerial stem tissue from seven-week-old plants was harvested, lyophilized, and used as a carbon source for bacterial growth analyses.

Wild-type lowland cultivar Alamo (commercial variety) switchgrass, sampled from Ardmore, Oklahoma, was provided by the Samuel Roberts Noble Foundation. Plants were harvested from an approximately 0.25-acre solid stand cut on 2 November 2007 and baled on 5 November 2007 (Brian Motes, Sr. Research Associate, Forage Improvement Division, The Samuel Roberts Noble Foundation to Susan Holladay, 26 September 2008). The material was shipped to NREL, where it was ground to pass through a 20-mesh screen. It was unwashed and air-dried at NREL to less than a 5% moisture content and then sieved to a -20/+80 particle size. Switchgrass and poplar (BESC, Poplar +80/-20, 2-2011) were obtained from Brian Davison, Oak Ridge National Laboratory, Oak Ridge, TN. Samples of plant biomass were used as received without any chemical or physical pretreatment and were not autoclaved. Sugars and soluble biomass used as carbon sources included: D-(+) maltose (Catalog No. M5895), pectin (P9135), and polygalacturonic acid (P1879), all from Sigma, (St. Louis, MO).

The reported cell density measurements are the average of three independent biological replicates for each carbon source after incubation at 75°C for 24 h in LOD medium [23]. All substrates were used at a final concentration of 5 g/liter. For growth media with maltose, cellobiose, pectin, and polygalacturonic acid, 20 μM uracil was added. The growth media with plant biomass (switchgrass, poplar, and Arabidopsis) did not contain added uracil. Each culture was subcultured in the respective carbon source two or three times in anaerobic serum bottles before inoculating a 50-mL batch culture for cell density measurements. Growth of *C. bescii* strains on maltose and cellobiose were measured by optical density using a Jenway Genova spectrophotometer at 680 nm. The growth of *C. bescii* strains in pectin, polygalacturonic acid, and the three biomass types (switchgrass, poplar, and Arabidopsis) were conducted using epifluorescence microscopy [9]. To estimate the numbers of cells per milliliter of sample, 9 mL of samples were fixed with 1 mL 37% (w/v) formaldehyde. The samples were shaken vigorously and kept at room temperature for 30 min, and then frozen at -20°C. A 0.8-mL aliquot of the fixed samples was stained with 0.2 mL of 0.1% (w/v) acridine orange and visualized with an Olympus BX40 microscope with an oil-immersion objective lens (Uplan F1 100X/1.3).

### Construction of the pectinase cluster deletion vector

A DNA fragment containing 1.08 kb of the 5’ flanking region and 1.0 kb of the 3’ flanking region of the pectinase gene cluster (Cbes1853-1856) was generated by overlap extension polymerase chain reaction (OE-PCR) using primers JF021, JF20.3, JF15.2, and JF014 with KpnI sites added to the 5’ end and an ApaLI site at the 3’ end. Wild-type *C. bescii* gDNA was used as a template. A 4.3-kb DNA fragment, containing an apramycin resistance gene cassette, the *pyrF* cassette [18], and sequences related to the *E. coli* pSC101 replication origin were amplified from pDCW88 [21] using primers DC081 (w/KpnI) and DC262 (w/ApaLI). The two linear DNA fragments were digested with KpnI and ApaLI and ligated to generate pJFW54 using a Fast-link DNA Ligase kit (Epicentre Biotechnologies, Madison, WI) according to the manufacturer’s instructions. The primers used in this construction are shown in Additional file 1: Table S2, and a detailed diagram of the vector is shown in Additional file 1: Figure S1. *E. coli* strain DH5α was transformed by electroporation in a 2-mm-gap cuvette at 2.5 V, and transformants were selected for apramycin resistance. The DNA sequence of the final vector was determined to confirm its structure (Macrogen, Cambridge, MA). All plasmids are available upon request.

### Transformation and 5-FOA selection

One microgram of M.CbeI methylated pJFW54 was electrotransformed into JWCB005 (*ΔpyrFA*) as described [18]. Recovery cultures after the electro-pulse were plated onto solid defined medium without uracil for selection of transformants. The transformants were inoculated into liquid medium for genomic DNA extraction and subsequent PCR screening of the target region to confirm the homologous recombination event using two primer sets of DC262/DC410 and DC230/DC411. The transformants were then inoculated into the defined medium without uracil overnight at 75°C, harvested by centrifugation (5,000 rpm for 15 min), and resuspended in 1 mL of 1 × base salt [10]. One hundred microliters of cell suspension was plated directly onto the defined medium plates containing 8 mM 5-FOA and 20 μM uracil as described [20]. Colonies resistant to 5-FOA were cultured in nonselective complex medium for genomic DNA isolation and subsequent PCR screening of the targeted region using primers JF204 and JF049. The JF204 primer is located outside the homologous regions used to construct the pectinase gene cluster deletion (Figure 1A). For PCR, the extension time utilized was sufficient to allow for amplification of the wild-type allele. Transformants containing the expected deletion were further purified by three additional passages on nonselective solid medium and screened by PCR to confirm the absence of the wild-type allele using primers DC409/DC410 to amplify Cbes1854, and DC411/DC412 to amplify a portion of Cbes1855.

### Sequential extraction and glycome profiling

Sequential plant cell wall extractions and glycome profiling of the different biomass samples were carried out as described previously [25,30]. Plant glycan-directed monoclonal antibodies [24] were from laboratory stocks (CCRC, JIM, and MAC series) at the Complex Carbohydrate Research Center (available through CarboSource Services; http://www.carbosource.net) or were obtained from BioSupplies (Australia) (BG1, LAMP). A description of the mAbs used in this study can be found in Additional file 1. Additional file 1: Table S1 includes links to a web database, Wall*MAb*DB (http://www.wallmabdb.net) that provides detailed information about each antibody.

## Additional file

Additional file 1: Figure S1. Diagram of the pectinase gene cluster (Cbes1853 - 1856) deletion vector. **Figure S2.** Microbial deconstruction of different sugar and plant biomass substrates by wild type and mutant C*aldicellulosiruptor bescii* following growth at 75°C for 24 hours. **Table S1.** Listing of plant cell wall glycan-directed monoclonal antibodies (mAbs) used for glycome profiling analyses (Figures 3, 4, 5, S3, and S4). **Table S2.** Primers used in this study. **Figure S3.** Glycome profiling of supernatants resulting from the growth media obtained before and after bacterial growth on arabidopsis biomass. **Figure S4.** Glycome profiling of supernatants resulting from the growth media obtained before and after bacterial growth switchgrass biomass.

## Abbreviations

5-FOA: 5-fluoroorotic acid
Ara: Arabidopsis
*C. bescii*: *Caldicellulosiruptor bescii*
CBM: carbohydrate-binding module
CBP: consolidated bioprocessing
LB broth: Luria-Bertani broth
LOD: low osmolarity defined
PG: polygalacturonic acid
PL: polysaccharide lyase
SG: switchgrass
YE: yeast extract

## References

[1] Chundawat SP, Beckham GT, Himmel ME, Dale BE (2011). Deconstruction of lignocellulosic biomass to fuels and chemicals. Annu Rev Chem Biomol Eng.

[2] Mohnen D, Bar-Peled M, Somerville C (2009). Cell Wall Polysaccharide Synthesis.

[3] Albersheim PDA, Roberts K, Sederoff R, Staehelin A (2011). Plant Cell Walls.

[4] Atmodjo MA, Sakuragi Y, Zhu X, Burrell AJ, Mohanty SS, Atwood JA, Orlando R, Scheller HV, Mohnen D (2011). Galacturonosyltransferase (GAUT)1 and GAUT7 are the core of a plant cell wall pectin biosynthetic homogalacturonan:galacturonosyltransferase complex. Proc Natl Acad Sci U S A.

[5] Yin Y, Chen H, Hahn MG, Mohnen D, Xu Y (2010). Evolution and function of the plant cell wall synthesis-related glycosyltransferase family 8. Plant Physiol.

[6] Lionetti V, Francocci F, Ferrari S, Volpi C, Bellincampi D, Galletti R, D’Ovidio R, De Lorenzo G, Cervone F (2010). Engineering the cell wall by reducing de-methyl-esterified homogalacturonan improves saccharification of plant tissues for bioconversion. Proc Natl Acad Sci U S A.

[7] Biswal AK, Soeno K, Gandla ML, Immerzeel P, Pattathil S, Lucenius J, Serimaa R, Hahn MG, Moritz T, Jonsson LJ, Israelsson-Nordström M, Mellerowicz EJ (2014). Aspen pectate lyase PtxtPL1-27 mobilizes matrix polysaccharides from woody tissues and improves saccharification yield. Biotechnol Biofuels.

[8] Tan L, Eberhard S, Pattathil S, Warder C, Glushka J, Yuan C, Hao Z, Zhu X, Avci U, Miller JS, Baldwin D, Pham C, Orlando R, Darvill A, Hahn MG, Kieliszewski MJ, Mohnen D (2013). An Arabidopsis cell wall proteoglycan consists of pectin and arabinoxylan covalently linked to an arabinogalactan protein. Plant Cell.

[9] Blumer-Schuette SE, Giannone RJ, Zurawski JV, Ozdemir I, Ma Q, Yin Y, Xu Y, Kataeva I, Poole FL, Adams MW, Hamilton-Brehm SD, Elkins JG, Larimer FW, Land ML, Hauser LJ, Cottingham RW, Hettich RL, Kelly RM (2012). *Caldicellulosiruptor* core and pangenomes reveal determinants for noncellulosomal thermophilic deconstruction of plant biomass. J Bacteriol.

[10] Yang SJ, Kataeva I, Hamilton-Brehm SD, Engle NL, Tschaplinski TJ, Doeppke C, Davis M, Westpheling J, Adams MW (2009). Efficient degradation of lignocellulosic plant biomass, without pretreatment, by the thermophilic anaerobe “*Anaerocellum thermophilum*” DSM 6725. Appl Environ Microbiol.

[11] Blumer-Schuette SE, Lewis DL, Kelly RM (2010). Phylogenetic, microbiological, and glycoside hydrolase diversities within the extremely thermophilic, plant biomass-degrading genus *Caldicellulosiruptor*. Appl Environ Microbiol.

[12] Kataeva I, Foston MB, Yang S-J, Pattathil S, Biswal AK, Poole Ii FL, Basen M, Rhaesa AM, Thomas TP, Azadi P, Olman V, Saffold TD, Mohler KE, Lewis DL, Doeppke C, Zeng Y, Tschaplinski TJ, York WS, Davis M (2013). Carbohydrate and lignin are simultaneously solubilized from unpretreated switchgrass by microbial action at high temperature. Energy Environ Sci.

[13] Dam P, Kataeva I, Yang SJ, Zhou F, Yin Y, Chou W, Poole FL, Westpheling J, Hettich R, Giannone R, Lewis DL, Kelly R, Gilbert HJ, Henrissat B, Xu Y, Adams MWW (2011). Insights into plant biomass conversion from the genome of the anaerobic thermophilic bacterium *Caldicellulosiruptor bescii* DSM 6725. Nucleic Acids Res.

[14] Zhang Z, Schwartz S, Wagner L, Miller W (2000). A greedy algorithm for aligning DNA sequences. J Comput Biol.

[15] Altschul SF, Madden TL, Schaffer AA, Zhang J, Zhang Z, Miller W, Lipman DJ (1997). Gapped BLAST and PSI-BLAST: a new generation of protein database search programs. Nucleic Acids Res.

[16] McKie VA, Vincken JP, Voragen AG, van den Broek LA, Stimson E, Gilbert HJ (2001). A new family of rhamnogalacturonan lyases contains an enzyme that binds to cellulose. Biochem J.

[17] Alahuhta M, Brunecky R, Chandrayan P, Kataeva I, Adams MW, Himmel ME, Lunin VV (2013). The structure and mode of action of *Caldicellulosiruptor bescii* family 3 pectate lyase in biomass deconstruction. Acta Crystallogr Sect D: Biol Crystallogr.

[18] Chung D, Cha M, Farkas J, Westpheling J (2013). Construction of a stable replicating shuttle vector for *Caldicellulosiruptor* species: use for extending genetic methodologies to other members of this genus. PLoS ONE.

[19] Cantarel BL, Coutinho PM, Rancurel C, Bernard T, Lombard V, Henrissat B (2009). The Carbohydrate-Active EnZymes database (CAZy): an expert resource for Glycogenomics. Nucleic Acids Res.

[20] Chung D, Farkas J, Huddleston JR, Olivar E, Westpheling J (2012). Methylation by a unique α-class N_4_-cytosine methyltransferase is required for DNA transformation of *Caldicellulosiruptor besci*i DSM6725. PLoS ONE.

[21] Chung D, Farkas J, Westpheling J (2013). Overcoming restriction as a barrier to DNA transformation in *Caldicellulosiruptor* species results in efficient marker replacement. Biotechnol Biofuels.

[22] Basen M, Rhaesa AM, Kataeva I, Prybol CJ, Scott IM, Poole FL, Adams MW (2014). Degradation of high loads of crystalline cellulose and of unpretreated plant biomass by the thermophilic bacterium *Caldicellulosiruptor bescii*. Bioresour Technol.

[23] Farkas J, Chung D, Cha M, Copeland J, Grayeski P, Westpheling J (2013). Improved growth media and culture techniques for genetic analysis and assessment of biomass utilization by *Caldicellulosiruptor bescii*. J Ind Microbiol Biotechnol.

[24] Pattathil S, Avci U, Baldwin D, Swennes AG, McGill JA, Popper Z, Bootten T, Albert A, Davis RH, Chennareddy C, Dong R, O'Shea B, Rossi R, Leoff C, Freshour G, Narra R, O'Neil M, York WS, Hahn MG (2010). A comprehensive toolkit of plant cell wall glycan-directed monoclonal antibodies. Plant Physiol.

[25] DeMartini JD, Pattathil S, Avci U, Szekalski K, Mazumder K, Hahn MG, Wyman CE (2011). Application of monoclonal antibodies to investigate plant cell wall deconstruction for biofuels production. Energy Environ Sci.

[26] Keegstra K, Talmadge KW, Bauer WD, Albersheim P (1973). The structure of plant cell walls: III.A model of the walls of suspension-cultured sycamore cells based on the interconnections of the macromolecular components. Plant Physiol.

[27] Bauer S, Vasu P, Persson S, Mort AJ, Somerville CR (2006). Development and application of a suite of polysaccharide-degrading enzymes for analyzing plant cell walls. Proc Natl Acad Sci U S A.

[28] Sambrook J, Russell D (2001). Molecular Cloning: A Laboratory Manual.

[29] Caffall KH, Pattathil S, Phillips SE, Hahn MG, Mohnen D (2009). *Arabidopsis thaliana* T-DNA mutants implicate GAUT genes in the biosynthesis of pectin and xylan in cell walls and seed testa. Mol Plant.

[30] Pattathil S, Avci U, Miller JS, Hahn MG (2012). Immunological approaches to plant cell wall and biomass characterization: glycome profiling. Methods Mol Biol.

