# Supplementary material for: Deletion of a gene cluster encoding pectin degrading enzymes in Caldicellulosiruptor bescii reveals an important role for pectin in plant biomass recalcitrance
Source: Biotechnol Biofuels. 2014 Oct 10;7:147. doi: 10.1186/s13068-014-0147-1 (PMC4198799; doi:10.1186/s13068-014-0147-1)
Supplement: Additional file 1: Figure S1. — Diagram of the pectinase gene cluster (Cbes1853 - 1856) deletion vector. Figure S2. Microbial deconstruction of different sugar and plant biomass substrates by wild type and mutant Caldicellulosiruptor bescii following growth at 75°C for 24 hours. Table S1. Listing of plant cell wall glycan-directed monoclonal antibodies (mAbs) used for glycome profiling analyses (Figures 3, 4, 5, S3, and S4). Table S2. Primers used in this study. Figure S3. Glycome profiling of supernatants resulting from the growth media obtained before and after bacterial growth on arabidopsis biomass. Figure S4. Glycome profiling of supernatants resulting from the growth media obtained before and after bacterial growth switchgrass biomass. [file 13068_2014_147_MOESM1_ESM.docx]

**Fig. S1. Diagram of the pectinase gene cluster (Cbes1853 - 1856) deletion vector.** The gray colored boxed sequences originated from *C. bescii*. Restriction sites and primers used for this construction are indicated. Apr, apramycin resistant gene cassette; pSC101, low copy origin of replication from *E. coli*; *repA*, a plasmid-encoded gene required for pSC101 replication; *par*, partition locus. The two kb flanking regions from up- and down-stream of the cluster for homologous recombination and the *pyrF* cassette for selection of uracil prototrophy are also indicated.

(A)

(B)

(C)

JWCB005

(*ΔpyrFA*)

JWCB010

(*ΔpyrFA*

*ΔCbes1853-1856*)

wild type

(D)


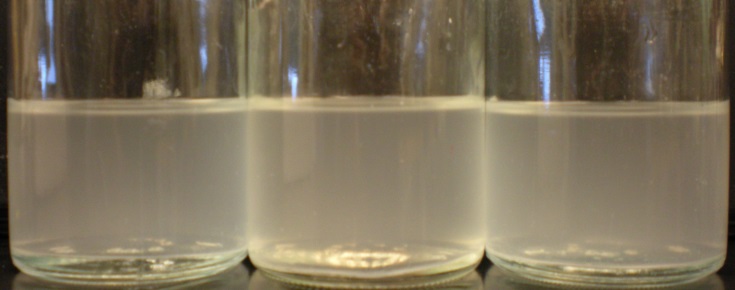

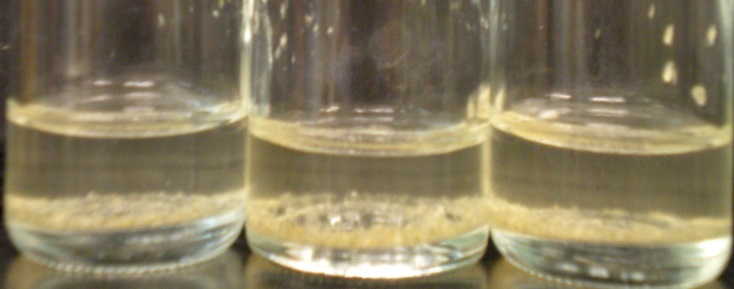

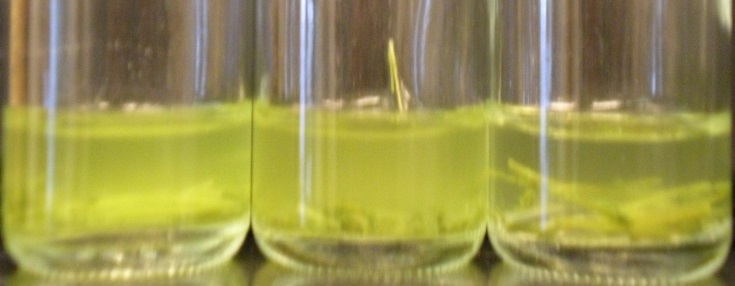

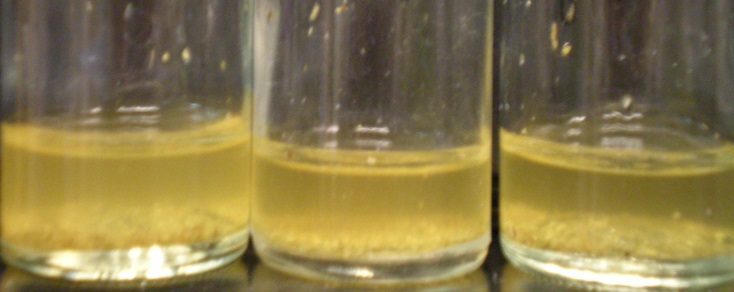


**Fig.S2. Microbial deconstruction of different sugar and plant biomass substrates by wild type and mutant C*aldicellulosiruptor bescii* following growth at 75 ̊C for 24 hours**. Culture vessel showing substrate remaining following growth of the wild type, the parent strain (JWCB005) and the pectinase gene cluster deletion mutant (JWCB010). *C. bescii* cells were grown at 75 ^o^C for 24 hours on (A) maltose, (B) dried arabidopsis aerial stem tissue, (C) switchgrass, or (D) Poplar.**Table S1.** Listing of plant cell wall glycan-directed monoclonal antibodies (mAbs) used for glycome profiling analyses (Figs 3, 4, 5, S3, and S4). The groupings of antibodies are based on a hierarchical clustering of ELISA data generated from a screen of all mAbs against a panel of plant polysaccharide preparations [[1](#_ENREF_1), [2](#_ENREF_2)] that groups the mAbs according to the predominant polysaccharides that they recognize. The majority of listings link to the Wall*Mab*DB plant cell wall monoclonal antibody database (<http://www.wallmabdb.net>) that provides detailed descriptions of each mAb, including immunogen, antibody isotype, epitope structure (to the extent known), supplier information, and related literature citations.

**Glycan Group Recognized mAb Names**

| Non-Fucosylated Xyloglucan-1 | [CCRC-M95](http://glycomics.ccrc.uga.edu/wall2/jsp/abdetails.jsp?abnumber=162&abname=CCRC-M95) |
| --- | --- |
|  | [CCRC-M101](http://glycomics.ccrc.uga.edu/wall2/jsp/abdetails.jsp?abnumber=163&abname=CCRC-M101) |
|  |  |
| Non-Fucosylated Xyloglucan-2 | [CCRC-M104](http://glycomics.ccrc.uga.edu/wall2/jsp/abdetails.jsp?abnumber=164&abname=CCRC-M104) |
|  | [CCRC-M89](http://glycomics.ccrc.uga.edu/wall2/jsp/abdetails.jsp?abnumber=160&abname=CCRC-M89) |
|  | [CCRC-M93](http://glycomics.ccrc.uga.edu/wall2/jsp/abdetails.jsp?abnumber=161&abname=CCRC-M93) |
|  | [CCRC-M87](http://glycomics.ccrc.uga.edu/wall2/jsp/abdetails.jsp?abnumber=158&abname=CCRC-M87) |
|  | [CCRC-M88](http://glycomics.ccrc.uga.edu/wall2/jsp/abdetails.jsp?abnumber=159&abname=CCRC-M88) |
|  |  |
| Non-Fucosylated Xyloglucan-3 | [CCRC-M100](http://glycomics.ccrc.uga.edu/wall2/jsp/abdetails.jsp?abnumber=114&abname=CCRC-M100) |
|  | [CCRC-M103](http://glycomics.ccrc.uga.edu/wall2/jsp/abdetails.jsp?abnumber=113&abname=CCRC-M103) |
|  |  |
| Non-Fucosylated Xyloglucan-4 | [CCRC-M58](http://glycomics.ccrc.uga.edu/wall2/jsp/abdetails.jsp?abnumber=155&abname=CCRC-M58) |
|  | [CCRC-M86](http://glycomics.ccrc.uga.edu/wall2/jsp/abdetails.jsp?abnumber=157&abname=CCRC-M86) |
|  | [CCRC-M55](http://glycomics.ccrc.uga.edu/wall2/jsp/abdetails.jsp?abnumber=148&abname=CCRC-M55) |
|  | [CCRC-M52](http://glycomics.ccrc.uga.edu/wall2/jsp/abdetails.jsp?abnumber=145&abname=CCRC-M52) |
|  | [CCRC-M99](http://glycomics.ccrc.uga.edu/wall2/jsp/abdetails.jsp?abnumber=152&abname=CCRC-M99) |
|  |  |
| Non-Fucosylated Xyloglucan-5 | [CCRC-M54](http://glycomics.ccrc.uga.edu/wall2/jsp/abdetails.jsp?abnumber=147&abname=CCRC-M54) |
|  | [CCRC-M48](http://glycomics.ccrc.uga.edu/wall2/jsp/abdetails.jsp?abnumber=77&abname=CCRC-M48) |
|  | [CCRC-M49](http://glycomics.ccrc.uga.edu/wall2/jsp/abdetails.jsp?abnumber=76&abname=CCRC-M49) |
|  | [CCRC-M96](http://glycomics.ccrc.uga.edu/wall2/jsp/abdetails.jsp?abnumber=151&abname=CCRC-M96) |
|  | [CCRC-M50](http://glycomics.ccrc.uga.edu/wall2/jsp/abdetails.jsp?abnumber=143&abname=CCRC-M50) |
|  | [CCRC-M51](http://glycomics.ccrc.uga.edu/wall2/jsp/abdetails.jsp?abnumber=144&abname=CCRC-M51) |
|  | [CCRC-M53](http://glycomics.ccrc.uga.edu/wall2/jsp/abdetails.jsp?abnumber=146&abname=CCRC-M53) |
|  |  |
| Non-Fucosylated Xyloglucan-6 | [CCRC-M57](http://glycomics.ccrc.uga.edu/wall2/jsp/abdetails.jsp?abnumber=154&abname=CCRC-M57) |
|  |  |
| Fucosylated Xyloglucan | [CCRC-M102](http://glycomics.ccrc.uga.edu/wall2/jsp/abdetails.jsp?abnumber=142&abname=CCRC-M102) |
|  | [CCRC-M39](http://glycomics.ccrc.uga.edu/wall2/jsp/abdetails.jsp?abnumber=78&abname=CCRC-M39) |
|  | [CCRC-M106](http://glycomics.ccrc.uga.edu/wall2/jsp/abdetails.jsp?abnumber=112&abname=CCRC-M106) |
|  | [CCRC-M84](http://glycomics.ccrc.uga.edu/wall2/jsp/abdetails.jsp?abnumber=124&abname=CCRC-M84) |
|  | [CCRC-M1](http://glycomics.ccrc.uga.edu/wall2/jsp/abdetails.jsp?abnumber=1&abname=CCRC-M1) |
|  |  |
|  |  |
| Xylan-1/XG | [CCRC-M111](http://glycomics.ccrc.uga.edu/wall2/jsp/abdetails.jsp?abnumber=168&abname=CCRC-M111) |
|  | [CCRC-M108](http://glycomics.ccrc.uga.edu/wall2/jsp/abdetails.jsp?abnumber=149&abname=CCRC-M108) |
|  | [CCRC-M109](http://glycomics.ccrc.uga.edu/wall2/jsp/abdetails.jsp?abnumber=150&abname=CCRC-M109) |
|  |  |
| Xylan-2 | [CCRC-M119](http://glycomics.ccrc.uga.edu/wall2/jsp/abdetails.jsp?abnumber=106&abname=CCRC-M119) |
|  | [CCRC-M115](http://glycomics.ccrc.uga.edu/wall2/jsp/abdetails.jsp?abnumber=110&abname=CCRC-M115) |
|  | [CCRC-M110](http://glycomics.ccrc.uga.edu/wall2/jsp/abdetails.jsp?abnumber=167&abname=CCRC-M110) |
|  | [CCRC-M105](http://glycomics.ccrc.uga.edu/wall2/jsp/abdetails.jsp?abnumber=165&abname=CCRC-M105) |
|  |  |
| Xylan-3 | [CCRC-M117](http://glycomics.ccrc.uga.edu/wall2/jsp/abdetails.jsp?abnumber=108&abname=CCRC-M117) |
|  | [CCRC-M113](http://glycomics.ccrc.uga.edu/wall2/jsp/abdetails.jsp?abnumber=171&abname=CCRC-M113) |
|  | [CCRC-M120](http://glycomics.ccrc.uga.edu/wall2/jsp/abdetails.jsp?abnumber=105&abname=CCRC-M120) |
|  | [CCRC-M118](http://glycomics.ccrc.uga.edu/wall2/jsp/abdetails.jsp?abnumber=107&abname=CCRC-M118) |
|  | [CCRC-M116](http://glycomics.ccrc.uga.edu/wall2/jsp/abdetails.jsp?abnumber=109&abname=CCRC-M116) |
|  | [CCRC-M114](http://glycomics.ccrc.uga.edu/wall2/jsp/abdetails.jsp?abnumber=111&abname=CCRC-M114) |
|  |  |
| Xylan-4 | CCRC-M154 |
|  | CCRC-M150 |
|  |  |
| Xylan-5 | CCRC-M144 |
|  | CCRC-M146 |
|  | CCRC-M145 |
|  | CCRC-M155 |
|  |  |
| Xylan-6 | CCRC-M153 |
|  | CCRC-M151 |
|  | CCRC-M148 |
|  | CCRC-M140 |
|  | CCRC-M139 |
|  | CCRC-M138 |
|  |  |
| Xylan-7 | CCRC-M160 |
|  | [CCRC-M137](http://glycomics.ccrc.uga.edu/wall2/jsp/abdetails.jsp?abnumber=173&abname=CCRC-M137) |
|  | CCRC-M152 |
|  | CCRC-M149 |
|  |  |
| Galactomannan-1 | [CCRC-M75](http://glycomics.ccrc.uga.edu/wall2/jsp/abdetails.jsp?abnumber=133&abname=CCRC-M75) |
|  | [CCRC-M70](http://glycomics.ccrc.uga.edu/wall2/jsp/abdetails.jsp?abnumber=61&abname=CCRC-M70) |
|  | [CCRC-M74](http://glycomics.ccrc.uga.edu/wall2/jsp/abdetails.jsp?abnumber=134&abname=CCRC-M74) |
|  |  |
| Galactomannan-2 | CCRC-M166 |
|  | CCRC-M168 |
|  | CCRC-M174 |
|  | CCRC-M175 |
| Acetylated Mannan | CCRC-M169 |
|  | CCRC-M170 |
|  |  |
| β-Glucan | [LAMP](http://glycomics.ccrc.uga.edu/wall2/jsp/abdetails.jsp?abnumber=47&abname=LAMP2H12H7) |
|  | [BG1](http://glycomics.ccrc.uga.edu/wall2/jsp/abdetails.jsp?abnumber=48&abname=BG1) |
|  |  |
| HG Backbone-1 | [CCRC-M131](http://glycomics.ccrc.uga.edu/wall2/jsp/abdetails.jsp?abnumber=181&abname=CCRC-M131) |
|  | [CCRC-M38](http://glycomics.ccrc.uga.edu/wall2/jsp/abdetails.jsp?abnumber=45&abname=CCRC-M38) |
|  | [JIM5](http://glycomics.ccrc.uga.edu/wall2/jsp/abdetails.jsp?abnumber=14&abname=JIM5) |
|  |  |
| HG Backbone-2 | [JIM136](http://glycomics.ccrc.uga.edu/wall2/jsp/abdetails.jsp?abnumber=57&abname=JIM136) |
|  | [JIM7](http://glycomics.ccrc.uga.edu/wall2/jsp/abdetails.jsp?abnumber=13&abname=JIM7) |
|  |  |
| RG-I Backbone | [CCRC-M69](http://glycomics.ccrc.uga.edu/wall2/jsp/abdetails.jsp?abnumber=172&abname=CCRC-M69) |
|  | [CCRC-M35](http://glycomics.ccrc.uga.edu/wall2/jsp/abdetails.jsp?abnumber=66&abname=CCRC-M35) |
|  | [CCRC-M36](http://glycomics.ccrc.uga.edu/wall2/jsp/abdetails.jsp?abnumber=37&abname=CCRC-M36) |
|  | [CCRC-M14](http://glycomics.ccrc.uga.edu/wall2/jsp/abdetails.jsp?abnumber=67&abname=CCRC-M14) |
|  | [CCRC-M129](http://glycomics.ccrc.uga.edu/wall2/jsp/abdetails.jsp?abnumber=104&abname=CCRC-M129) |
|  | [CCRC-M72](http://glycomics.ccrc.uga.edu/wall2/jsp/abdetails.jsp?abnumber=135&abname=CCRC-M72) |
|  |  |
| Linseed Mucilage RG-I | [JIM3](http://glycomics.ccrc.uga.edu/wall2/jsp/abdetails.jsp?abnumber=79&abname=JIM1) |
|  | [CCRC-M40](http://glycomics.ccrc.uga.edu/wall2/jsp/abdetails.jsp?abnumber=83&abname=CCRC-M40) |
|  | CCRC-M161 |
|  | CCRC-M164 |
|  |  |
| Physcomitrella Pectin | [CCRC-M98](http://glycomics.ccrc.uga.edu/wall2/jsp/abdetails.jsp?abnumber=115&abname=CCRC-M98) |
|  | [CCRC-M94](http://glycomics.ccrc.uga.edu/wall2/jsp/abdetails.jsp?abnumber=118&abname=CCRC-M94) |
|  |  |
| RG-Ia | [CCRC-M5](http://glycomics.ccrc.uga.edu/wall2/jsp/abdetails.jsp?abnumber=81&abname=CCRC-M5) |
|  | [CCRC-M2](http://glycomics.ccrc.uga.edu/wall2/jsp/abdetails.jsp?abnumber=8&abname=CCRC-M2) |
|  |  |
| RG-Ib | [JIM137](http://glycomics.ccrc.uga.edu/wall2/jsp/abdetails.jsp?abnumber=58&abname=JIM137) |
|  | [JIM101](http://glycomics.ccrc.uga.edu/wall2/jsp/abdetails.jsp?abnumber=55&abname=JIM101) |
|  | [CCRC-M61](http://glycomics.ccrc.uga.edu/wall2/jsp/abdetails.jsp?abnumber=138&abname=CCRC-M61) |
|  | [CCRC-M30](http://glycomics.ccrc.uga.edu/wall2/jsp/abdetails.jsp?abnumber=33&abname=CCRC-M30) |
|  |  |
| RG-Ic | [CCRC-M23](http://glycomics.ccrc.uga.edu/wall2/jsp/abdetails.jsp?abnumber=92&abname=CCRC-M23) |
|  | [CCRC-M17](http://glycomics.ccrc.uga.edu/wall2/jsp/abdetails.jsp?abnumber=74&abname=CCRC-M17) |
|  | [CCRC-M19](http://glycomics.ccrc.uga.edu/wall2/jsp/abdetails.jsp?abnumber=0&abname=CCRC-M19) |
|  | [CCRC-M18](http://glycomics.ccrc.uga.edu/wall2/jsp/abdetails.jsp?abnumber=0&abname=CCRC-M18) |
|  | [CCRC-M56](http://glycomics.ccrc.uga.edu/wall2/jsp/abdetails.jsp?abnumber=141&abname=CCRC-M56) |
|  | [CCRC-M16](http://glycomics.ccrc.uga.edu/wall2/jsp/abdetails.jsp?abnumber=73&abname=CCRC-M16) |
|  |  |
|  |  |
| RG-I/Arabinogalactan | [CCRC-M60](http://glycomics.ccrc.uga.edu/wall2/jsp/abdetails.jsp?abnumber=139&abname=CCRC-M60) |
|  | [CCRC-M41](http://glycomics.ccrc.uga.edu/wall2/jsp/abdetails.jsp?abnumber=82&abname=CCRC-M41) |
|  | [CCRC-M80](http://glycomics.ccrc.uga.edu/wall2/jsp/abdetails.jsp?abnumber=128&abname=CCRC-M80) |
|  | [CCRC-M79](http://glycomics.ccrc.uga.edu/wall2/jsp/abdetails.jsp?abnumber=129&abname=CCRC-M79) |
|  | [CCRC-M44](http://glycomics.ccrc.uga.edu/wall2/jsp/abdetails.jsp?abnumber=68&abname=CCRC-M44) |
|  | [CCRC-M33](http://glycomics.ccrc.uga.edu/wall2/jsp/abdetails.jsp?abnumber=75&abname=CCRC-M33) |
|  | [CCRC-M32](http://glycomics.ccrc.uga.edu/wall2/jsp/abdetails.jsp?abnumber=35&abname=CCRC-M32) |
|  | [CCRC-M13](http://glycomics.ccrc.uga.edu/wall2/jsp/abdetails.jsp?abnumber=43&abname=CCRC-M13) |
|  | [CCRC-M42](http://glycomics.ccrc.uga.edu/wall2/jsp/abdetails.jsp?abnumber=86&abname=CCRC-M42) |
|  | [CCRC-M24](http://glycomics.ccrc.uga.edu/wall2/jsp/abdetails.jsp?abnumber=93&abname=CCRC-M24) |
|  | [CCRC-M12](http://glycomics.ccrc.uga.edu/wall2/jsp/abdetails.jsp?abnumber=71&abname=CCRC-M12) |
|  | [CCRC-M7](http://glycomics.ccrc.uga.edu/wall2/jsp/abdetails.jsp?abnumber=3&abname=CCRC-M7) |
|  | [CCRC-M77](http://glycomics.ccrc.uga.edu/wall2/jsp/abdetails.jsp?abnumber=131&abname=CCRC-M77) |
|  | [CCRC-M25](http://glycomics.ccrc.uga.edu/wall2/jsp/abdetails.jsp?abnumber=84&abname=CCRC-M25) |
|  | [CCRC-M9](http://glycomics.ccrc.uga.edu/wall2/jsp/abdetails.jsp?abnumber=69&abname=CCRC-M9) |
|  | [CCRC-M128](http://glycomics.ccrc.uga.edu/wall2/jsp/abdetails.jsp?abnumber=183&abname=CCRC-M128) |
|  | [CCRC-M126](http://glycomics.ccrc.uga.edu/wall2/jsp/abdetails.jsp?abnumber=184&abname=CCRC-M126) |
|  | [CCRC-M134](http://glycomics.ccrc.uga.edu/wall2/jsp/abdetails.jsp?abnumber=102&abname=CCRC-M134) |
|  | [CCRC-M125](http://glycomics.ccrc.uga.edu/wall2/jsp/abdetails.jsp?abnumber=185&abname=CCRC-M125) |
|  | [CCRC-M123](http://glycomics.ccrc.uga.edu/wall2/jsp/abdetails.jsp?abnumber=187&abname=CCRC-M123) |
|  | [CCRC-M122](http://glycomics.ccrc.uga.edu/wall2/jsp/abdetails.jsp?abnumber=188&abname=CCRC-M122) |
|  | [CCRC-M121](http://glycomics.ccrc.uga.edu/wall2/jsp/abdetails.jsp?abnumber=189&abname=CCRC-M121) |
|  | [CCRC-M112](http://glycomics.ccrc.uga.edu/wall2/jsp/abdetails.jsp?abnumber=169&abname=CCRC-M112) |
|  | [CCRC-M21](http://glycomics.ccrc.uga.edu/wall2/jsp/abdetails.jsp?abnumber=88&abname=CCRC-M21) |
|  | [JIM131](http://glycomics.ccrc.uga.edu/wall2/jsp/abdetails.jsp?abnumber=94&abname=JIM131) |
|  | [CCRC-M22](http://glycomics.ccrc.uga.edu/wall2/jsp/abdetails.jsp?abnumber=46&abname=CCRC-M22) |
|  | [JIM132](http://glycomics.ccrc.uga.edu/wall2/jsp/abdetails.jsp?abnumber=56&abname=JIM132) |
|  | [JIM1](http://glycomics.ccrc.uga.edu/wall2/jsp/abdetails.jsp?abnumber=79&abname=JIM1) |
|  | [CCRC-M15](http://glycomics.ccrc.uga.edu/wall2/jsp/abdetails.jsp?abnumber=72&abname=CCRC-M15) |
|  | [CCRC-M8](http://glycomics.ccrc.uga.edu/wall2/jsp/abdetails.jsp?abnumber=29&abname=CCRC-M8) |
|  | [JIM16](http://glycomics.ccrc.uga.edu/wall2/jsp/abdetails.jsp?abnumber=62&abname=JIM16) |
|  |  |
| Arabinogalactan-1 | [JIM93](http://glycomics.ccrc.uga.edu/wall2/jsp/abdetails.jsp?abnumber=117&abname=JIM93) |
|  | [JIM94](http://glycomics.ccrc.uga.edu/wall2/jsp/abdetails.jsp?abnumber=95&abname=JIM94) |
|  | [JIM11](http://glycomics.ccrc.uga.edu/wall2/jsp/abdetails.jsp?abnumber=41&abname=JIM11) |
|  | [MAC204](http://glycomics.ccrc.uga.edu/wall2/jsp/abdetails.jsp?abnumber=23&abname=MAC204) |
|  | [JIM20](http://glycomics.ccrc.uga.edu/wall2/jsp/abdetails.jsp?abnumber=91&abname=JIM20) |
|  |  |
| Arabinogalactan-2 | [JIM14](http://glycomics.ccrc.uga.edu/wall2/jsp/abdetails.jsp?abnumber=31&abname=JIM14) |
|  | [JIM19](http://glycomics.ccrc.uga.edu/wall2/jsp/abdetails.jsp?abnumber=44&abname=JIM19) |
|  | [JIM12](http://glycomics.ccrc.uga.edu/wall2/jsp/abdetails.jsp?abnumber=191&abname=JIM12) |
|  | [CCRC-M133](http://glycomics.ccrc.uga.edu/wall2/jsp/abdetails.jsp?abnumber=96&abname=JIM133) |
|  | [CCRC-M107](http://glycomics.ccrc.uga.edu/wall2/jsp/abdetails.jsp?abnumber=166&abname=CCRC-M107) |
|  |  |
| Arabinogalactan-3 | [JIM4](http://glycomics.ccrc.uga.edu/wall2/jsp/abdetails.jsp?abnumber=40&abname=JIM4) |
|  | [CCRC-M31](http://glycomics.ccrc.uga.edu/wall2/jsp/abdetails.jsp?abnumber=34&abname=CCRC-M31) |
|  | [JIM17](http://glycomics.ccrc.uga.edu/wall2/jsp/abdetails.jsp?abnumber=39&abname=JIM17) |
|  | [CCRC-M26](http://glycomics.ccrc.uga.edu/wall2/jsp/abdetails.jsp?abnumber=85&abname=CCRC-M26) |
|  | [JIM15](http://glycomics.ccrc.uga.edu/wall2/jsp/abdetails.jsp?abnumber=32&abname=JIM15) |
|  | [JIM8](http://glycomics.ccrc.uga.edu/wall2/jsp/abdetails.jsp?abnumber=80&abname=JIM8) |
|  | [CCRC-M85](http://glycomics.ccrc.uga.edu/wall2/jsp/abdetails.jsp?abnumber=121&abname=CCRC-M85) |
|  | [CCRC-M81](http://glycomics.ccrc.uga.edu/wall2/jsp/abdetails.jsp?abnumber=127&abname=CCRC-M81) |
|  | [MAC266](http://glycomics.ccrc.uga.edu/wall2/jsp/abdetails.jsp?abnumber=98&abname=MAC266) |
|  | [PN 16.4B4](http://glycomics.ccrc.uga.edu/wall2/jsp/abdetails.jsp?abnumber=11&abname=PN%2016.4B4) |
|  |  |
| Arabinogalactan-4 | [MAC207](http://glycomics.ccrc.uga.edu/wall2/jsp/abdetails.jsp?abnumber=22&abname=MAC207) |
|  | [JIM133](http://glycomics.ccrc.uga.edu/wall2/jsp/abdetails.jsp?abnumber=96&abname=JIM133) |
|  | [JIM13](http://glycomics.ccrc.uga.edu/wall2/jsp/abdetails.jsp?abnumber=30&abname=JIM13) |
|  | [CCRC-M92](http://glycomics.ccrc.uga.edu/wall2/jsp/abdetails.jsp?abnumber=119&abname=CCRC-M92) |
|  | [CCRC-M91](http://glycomics.ccrc.uga.edu/wall2/jsp/abdetails.jsp?abnumber=120&abname=CCRC-M91) |
|  | [CCRC-M78](http://glycomics.ccrc.uga.edu/wall2/jsp/abdetails.jsp?abnumber=130&abname=CCRC-M78) |
|  |  |
| Unidentified | [MAC265](http://glycomics.ccrc.uga.edu/wall2/jsp/abdetails.jsp?abnumber=97&abname=MAC265) |
|  | [CCRC-M97](http://glycomics.ccrc.uga.edu/wall2/jsp/abdetails.jsp?abnumber=116&abname=CCRC-M97) |
|  |  |

Table S2. Primers used in this study.

| Primers | Sequences (5’ to 3’) | Description |
| --- | --- | --- |
| DC081 forward | AGAGAGGTACCACCAGCCTAACTTCGATCATTGGA | To amplify the vector DNA fragment to construct pJFW54 |
| DC262 reverse | TGTGTGGTGCACTCTGACGCTCAGTGGAACGAA | To amplify the vector DNA fragment to construct pJFW54 |
| DC230 reverse | AAGAGACGTCTCATCTGTGCATATGGACAG | To confirm the integration event for transformant |
| DC409 forward | ACATAGTTTGTGCTGTTCTGA | To amplify 2.18 kb fragment of genome region includes Cbes1854 |
| DC410 reverse | TGAAGAAGGTACACACGTGT | To amplify 2.18 kb fragment of genome region includes Cbes1854 |
| DC411 forward | TCAGATGAACCAGTATATGCTCT | To amplify 1.31 kb fragment of genome region includes the portion of Cbes1855 and Cbes1856 |
| DC412 reverse | ACAGAGAACGTTGAGAATGGCA | To amplify 1.31 kb fragment of genome region includes the portion of Cbes1855 and Cbes1856 |
| JF014 forward | AGTGGTACCTGGATTCCAGGCATGCTCGATG | To amplify 1.08 kb of 5’ flanking region of the Cbes1853 |
| JF15.2 reverse | ACATATGGTTCTCTATATATCATGTTCATACATATGGTTCTTCCCATTTTCCTACA | To amplify 1.08 kb of 5’ flanking region of the Cbes1853 |
| JF20.3 forward | TCAACAAATTTTTGATGCAGACCGACCTCCTTCTTGGTAAGAATC | To amplify 1.0 kb of 3’ flanking region of the Cbes1856 |
| JF021 reverse | TCTTGAGTGCACTCCATTCTACACTACAGAAGGCTTCT | To amplify 1.0 kb of 3’ flanking region of the Cbes1856 |
| JF204 forward | TGGTCAAATACTATCTCTGCA | To amplify the pectinase gene cluster region (Cbes1853-1856) |
| JF049 reverse | TGGAGTTAAGTTTATAGACATAAGGAG | To amplify the pectinase gene cluster region (Cbes1853-1856) |

***
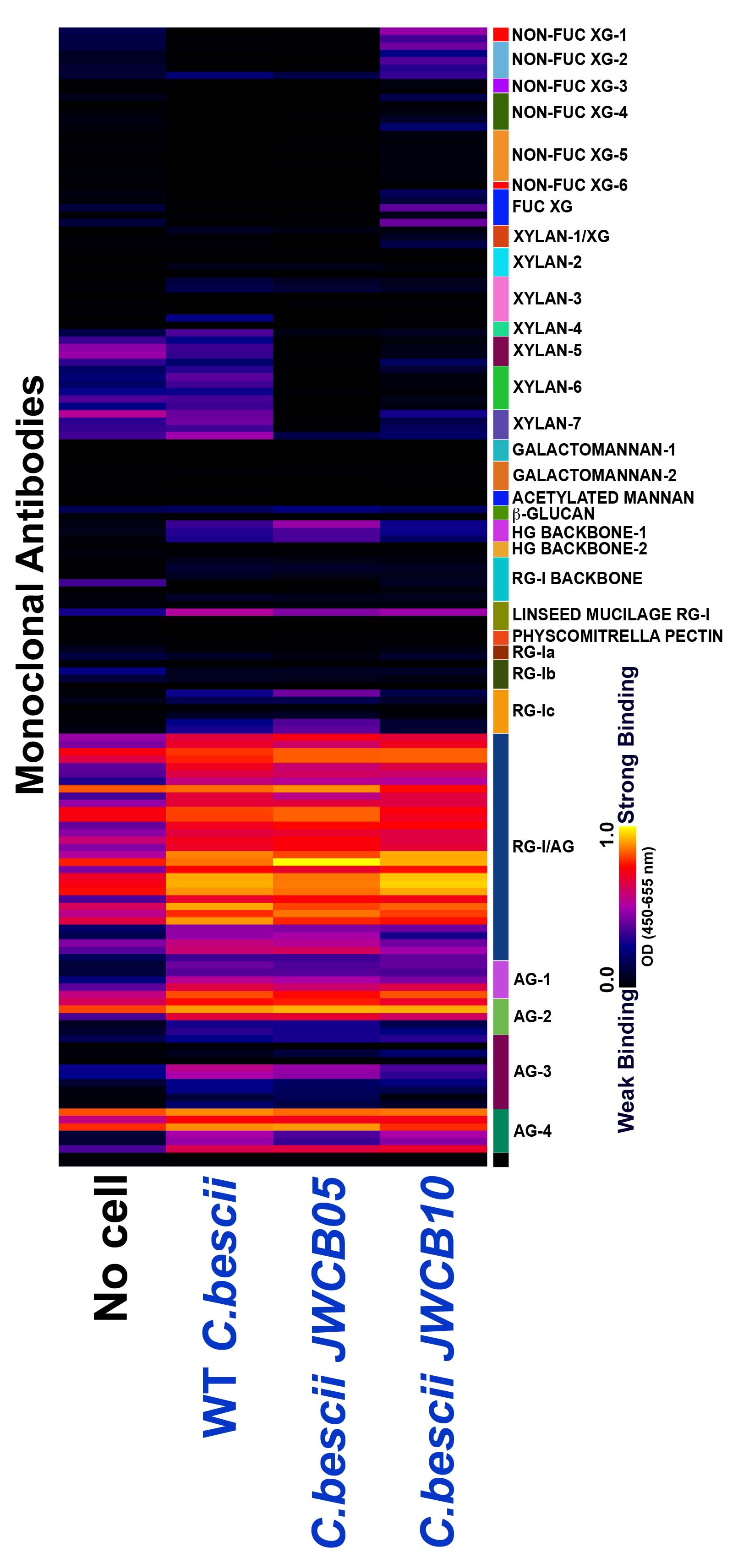
***

**Fig. S3. Glycome profiling of supernatants resulting from the growth media obtained before and after bacterial growth on arabidopsis biomass.** Samples were incubated at 75 ^o^C for 24 hr in the absence and presence of wild type, JWCB005 (*ΔpyrFA*) and JWCB010 (*ΔpyrFA ΔpecABCR).* The supernatant from the growth medium was ELISA screened using 155 mAbs directed against most major plant cell wall glycans (Table S1). The resulting binding response data are represented as heatmaps with white-red-dark blue scale indicating the strength of the ELISA signal (white, red and dark-blue colors depict strong, medium, and no binding, respectively). The mAbs are grouped based on the cell wall glycans they recognize as depicted in the panel at right hand side of the figure.

**
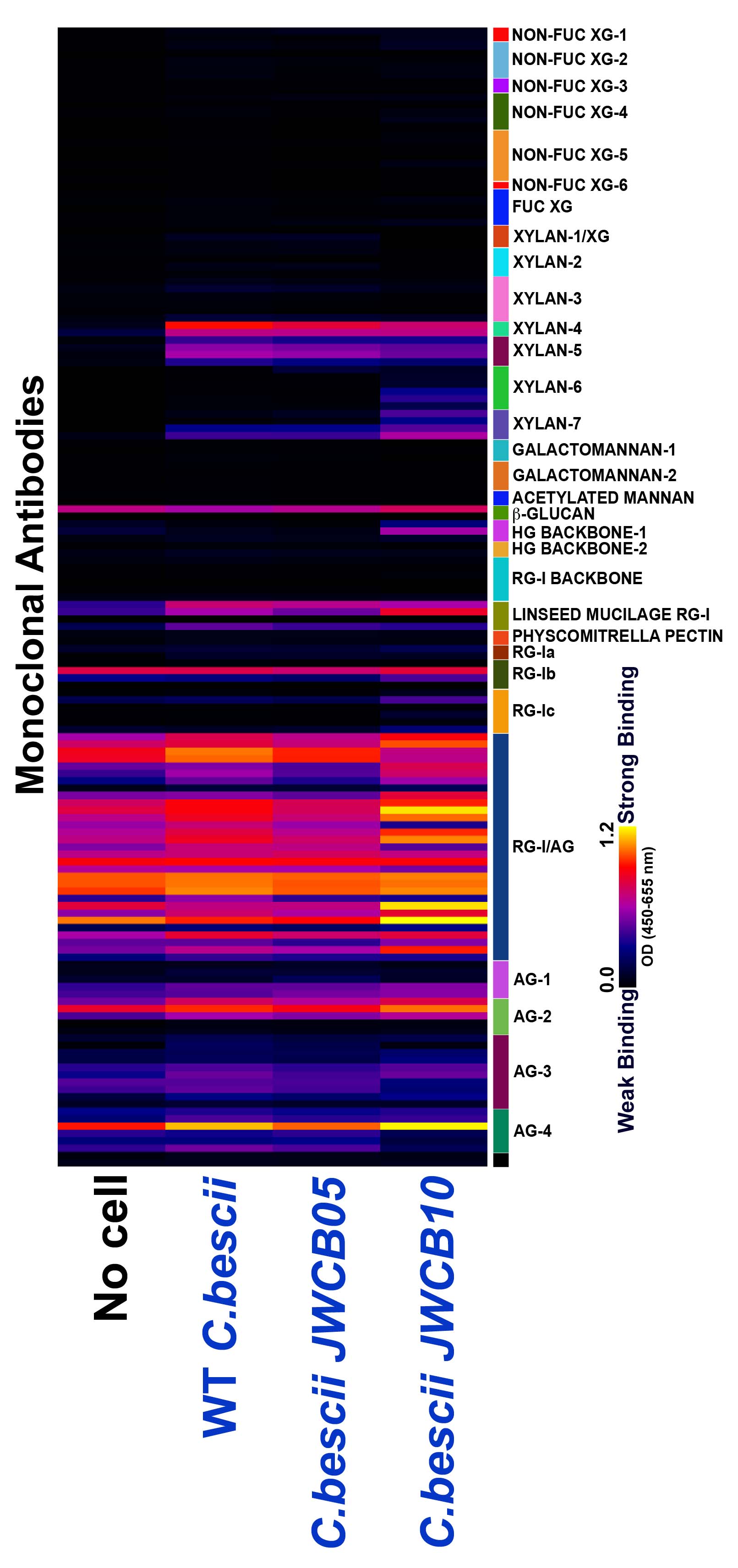
**

**Fig. S4. Glycome profiling of supernatants resulting from the growth media obtained before and after bacterial growth switchgrass biomass.** Samples were incubated at 75 ^o^C for 24 hr in the absence and presence of wild type, JWCB005 (*ΔpyrFA*) and JWCB010 (*ΔpyrFA ΔpecABCR).* The supernatant from the growth medium was ELISA screened using 155 mAbs directed against most major plant cell wall glycans (Table S1). The resulting binding response data are represented as heatmaps with white-red-dark blue scale indicating the strength of the ELISA signal (white, red and dark-blue colors depict strong, medium, and no binding, respectively). The mAbs are grouped based on the cell wall glycans they recognize as depicted in the panel at right hand side of the figure.

References

1. Pattathil S, Avci U, Baldwin D, Swennes AG, McGill JA, Popper Z, Bootten T, Albert A, Davis RH, Chennareddy C, et al: **A comprehensive toolkit of plant cell wall glycan-directed monoclonal antibodies.** *Plant physiology* 2010, **153:**514-525.

2. Pattathil S, Avci U, Miller JS, Hahn MG: **Immunological approaches to plant cell wall and biomass characterization: Glycome Profiling.** *Methods Mol Biol* 2012, **908:**61-72.
